# Corneal Absorption of a New Riboflavin-Nanostructured System for Transepithelial Collagen Cross-Linking

**DOI:** 10.1371/journal.pone.0066408

**Published:** 2013-06-13

**Authors:** Katia M. Bottos, Anselmo G. Oliveira, Patrícia A. Bersanetti, Regina F. Nogueira, Acácio A. S. Lima-Filho, José A. Cardillo, Paulo Schor, Wallace Chamon

**Affiliations:** 1 Department of Ophthalmology, Paulista School of Medicine, Federal University of Sao Paulo - UNIFESP, Sao Paulo, SP, Brazil; 2 Department of Pharmaceutics, School of Pharmaceutical Sciences, Sao Paulo State University, UNESP, Araraquara, SP, Brazil; 3 Department of Informatics in Health, Paulista School of Medicine, Federal University of Sao Paulo - UNIFESP, Sao Paulo, SP, Brazil; University of Reading, United Kingdom

## Abstract

Corneal collagen cross-linking (CXL) has been described as a promising therapy for keratoconus. According to standard CXL protocol, epithelium should be debrided before treatment to allow penetration of riboflavin into the corneal stroma. However, removal of the epithelium can increase procedure risks. In this study we aim to evaluate stromal penetration of a biocompatible riboflavin-based nanoemulsion system (riboflavin-5-phosphate and riboflavin-base) in rabbit corneas with intact epithelium. Two riboflavin nanoemulsions were developed. Transmittance and absorption coefficient were measured on corneas with intact epithelia after 30, 60, 120, 180, and 240 minutes following exposure to either the nanoemulsions or standard 0.1% or 1% riboflavin-dextran solutions. For the nanoemulsions, the epithelium was removed after measurements to assure that the riboflavin had passed through the hydrophobic epithelium and retained within the stroma. Results were compared to de-epithelialized corneas exposed to 0.1% riboflavin solution and to the same riboflavin nanoemulsions for 30 minutes (standard protocol). Mean transmittance and absorption measured in epithelialized corneas receiving the standard 0.1% riboflavin solution did not reach the levels found on the debrided corneas using the standard technique. Neither increasing the time of exposure nor the concentration of the riboflavin solution from 0.1% to 1% improved riboflavin penetration through the epithelium. When using riboflavin-5-phosphate nanoemulsion for 240 minutes, we found no difference between the mean absorption coefficients to the standard cross-linking protocol (*p* = 0.54). Riboflavin nanoemulsion was able to penetrate the corneal epithelium, achieving, after 240 minutes, greater stromal concentration when compared to debrided corneas with the standard protocol (*p* = 0.002). The riboflavin-5-phosphate nanoemulsion diffused better into the stroma than the riboflavin-base nanoemulsion.

## Introduction

Corneal collagen cross-linking (CXL) has emerged as a promising and innovative treatment for progressive keratoconus and surgically-related ectasia [1]. Recently, a significant number of studies have used this treatment for a wider range of applications, including microbial keratitis [2] and prevention of keratectasia prior to refractive surgery [3]. The standard CXL protocol proposed by Wollensak et al. recommended removal of the epithelium before treatment to allow penetration of riboflavin into the stroma [1]. Experimental and clinical investigations have shown that the intact epithelium does not block the effect of ultraviolet A (UVA) light [4], but decreases the effectiveness of the treatment by impairing the adequate stromal diffusion of riboflavin [5]–[11]. However, de-epithelialization is not risk-free and can increase the rate of corneal infections, haze, scarring, and infiltrates [12]–[16]. Moreover, it can cause postoperative pain, photophobia, and delayed visual rehabilitation [1]. Some modifications of the initial treatment have been suggested to overcome these disadvantages. Studies reported the use of benzalkonium chloride (BAK) to loosen tight junctions of corneal epithelial cells and facilitate riboflavin stromal diffusion [9], [17]. However, both *in vitro* and *in vivo* studies have demonstrated adverse effects of BAK on epithelial cell populations [18]–[21]. Recently, BAK-induced corneal neurotoxicity has been demonstrated [21]. Riboflavin in association with anesthetic drops poorly penetrated the cornea [7], and even superficial epithelial trauma was not sufficient to permit the adequate penetration of riboflavin into the stroma. Samaras et al. confirmed these findings not being able to increase penetration of riboflavin by using 20% alcohol in one group, and a grid pattern epithelial debridement in another group [6]. Neither groups had an acceptable and homogeneous riboflavin penetration. Furthermore, prolonging the exposure time to standard hydrophilic riboflavin did not lead to increased stromal saturation in corneas with intact epithelium [22]. Other alternatives to avoid complete epithelial debridement include a central corneal pocket created by a femtosecond laser that allows for direct intrastromal drug administration [23], [24]. However, one should be concerned regarding biomechanical stability after stromal dissection by the femtosecond laser. A riboflavin complex, with edetate disodium (EDTA) and tromethamine, has been tested for transepithelial CXL. However, due to its limited permeation through the epithelium, the CXL effect was reduced [25]. Recently, cyclodextrins were successfully used to enhance riboflavin solubility in water and to improve its permeability through fresh and cryopreserved bovine corneas [26]. In summary, none of the present modalities has proven to be clinically effective in delivering an adequate intrastromal concentration of riboflavin, hence a better method still needs to be developed.

Formulation is the most important factor in facilitating penetration of riboflavin through the epithelium. The standard riboflavin solution (10 mg riboflavin-5-phosphate in 10 mL 20% dextran T-500) has hydrophilic characteristics that prevent full diffusion of this drug through the lipophilic epithelium. Moreover, the electrostatic repulsion between the anionic riboflavin-5-phosphate and the negatively charged surface of the cornea further reduces its epithelial penetration.

Considering physicochemical properties, both, riboflavin-base and riboflavin-5-phosphate have no ability to permeate the cornea epithelium and achieve access to the stroma. Due to its lipophilic characteristic, riboflavin-base interacts with the epithelium and do not achieve adequate concentration into stroma. On the other hand, due to both hydrophilic property and the anionic character that is strongly repelled by the cornea epithelium, the hydrophilic riboflavin-5-phosphate does not sufficiently permeate to the stroma [27]. Thus, an appropriated vehicle should associate both bioadhesive and amphiphilic properties to allow the riboflavin to penetrate the epithelium and diffuse into the corneal hydrophilic stroma. In the present study we evaluated stromal absorption of two different BAK-free, amphiphilic riboflavin-based nanoemulsions (riboflavin-5-phosphate and riboflavin-base) in rabbit corneas with intact epithelium.

## Materials and Methods

### Ethics Statement

All procedures were carried out in accordance with the institutional guideline for the care and use of laboratory animals at the Federal University of São Paulo. The experimental protocol was approved by the Ethics in Research Committee of the Federal University of São Paulo, under the number 1565/07.

### Riboflavin Ocular Formulations

Four riboflavin formulations were tested in this study:

- 0.1% riboflavin-5-phosphate solution: A standard hydrophilic riboflavin in dextran was prepared. This is the same formulation used routinely in the standard CXL procedure. The solution is comprised of 10 mg riboflavin-5-phosphate dissolved in 10 mL 20% dextran T-500 (400 mOsm/mL).
- 1% riboflavin-5-phosphate solution: A concentrated hydrophilic riboflavin in dextran was prepared. This solution is comprised of an increased concentration of riboflavin-5-phosphate (in 20% dextran T-500) to study the effect of riboflavin concentration on the absorbance of epithelialized corneas.
- 0.5% riboflavin-5-phosphate nanoemulsion: Nanoemulsions containing soy phosphatidylcholine (SPC) (12% w/w), polyoxyethylene sorbitan monolaurate (Tween) (12% w/w) and ethylene oxide/propylene oxide block copolymer (Pluronic) (2% w/w) as surfactant; propylene glycol dicaprylate/dicaprate (5% w/w) (Captex) as oil phase; stearylamine (0.3% w/w) as cationic agent; saline as aqueous phase (68,2% w/w) and riboflavin-5-phosphate (0.5% w/w) were obtained by sonication. For preparation SPC, Tween, Pluronic and stearylamine were completely homogenized with the oil phase Captex. Riboflavin-5-phosphate was dissolved in the aqueous phase. The aqueous phase containing riboflavin-5-phosphate was added to the lipid phase by vortexing the mixture, and treated with ultrasound irradiation for up to 15 minutes using a probe-tip sonicator (600 watts of nominal power) using 1 minute pulses and 1 minute intervals.
- 0.5% riboflavin-base nanoemulsion: Nanoemulsions were prepared in a similar manner by sonication using riboflavin-base. SPC, Tween, Pluronic, stearylamine and riboflavin-base were completely homogenized with the oil phase Captex. The aqueous phase was added to the lipid phase by vortexing the mixture, and treated with ultrasound irradiation for up to 15 minutes using a probe-tip sonicator (600 watts of nominal power) using 1-minute pulses and 1-minute intervals.

Nanoemulsions can be defined as submicron sized oil-in-water (o/w) emulsions with mean droplet diameters usually ranging from 10 to 100 nm [28]. These colloidal systems show a translucent appearance and have been used for different applications in the pharmaceutical fields [29], [30]. They are prepared by high-energy transfer processes, such as ultrasound irradiation and high-pressure homogenization and do not show a separation phase during storage [31].

Different interactions can occur with cationic nanodroplets depending on the ionic specie of riboflavin tested. Neutral riboflavin-base predominantly interacts by hydrophobic effect with the nanoemulsion oil core [32]. On the other hand, the anionic riboflavin-5-phosphate primarily interacts by electrostatic effect with the positively charged droplets of nanoemulsions, and also by the hydrophobic contribution of the non-ionic region of the molecule [33].

For drug-unloaded nanoemulsions used in this study, the light scattering measurements of the diameters, polydispersity index and zeta potential are shown in Table 1. Regardless the presence of the riboflavin, the obtained diameters values demonstrate that the nanodroplets were efficiently formed with the stabilizing agents used in the formulations. The polydispersity index less than 0.3 indicates excellent homogeneity in the droplets diameters distribution. Furthermore, the values obtained for the zeta potential in the range of 30–40 mV are indicative of excellent dispersion of the nanodroplets in the bulk vehicle. However, when riboflavin was incorporated into nanoemulsions the mean diameter increased to about 78 nm for the riboflavin-5-phosphate and 82 nm for riboflavin-base, both with polydispersity index less than 0.3 (Table 1). This increase in diameter of the nanodroplets shows a swelling of the local volume of the dispersed oil phase demonstrating that riboflavin was incorporated into nanoemulsions. Increases of the local volume of oil droplet are a common feature in drug delivery from colloidal systems such as micro- and nanoemulsions [34], [35].

**Table 1:** Diameter of nanoemulsion after preparation.

Sample	Diameter (nm) Mean (n = 10)	Polidispersity Index	Zeta Potential (mV)
Unloaded nanoemulsion	67.8	0.254	+43.1
Riboflavin-5-loaded nanoemulsion	78.3	0.274	+31.3

The systems remained stable, optically semi-transparent and no phase separation or solid sediment was observed after centrifugation 8,500 (× g) for 15 min.

**Figure 1:**
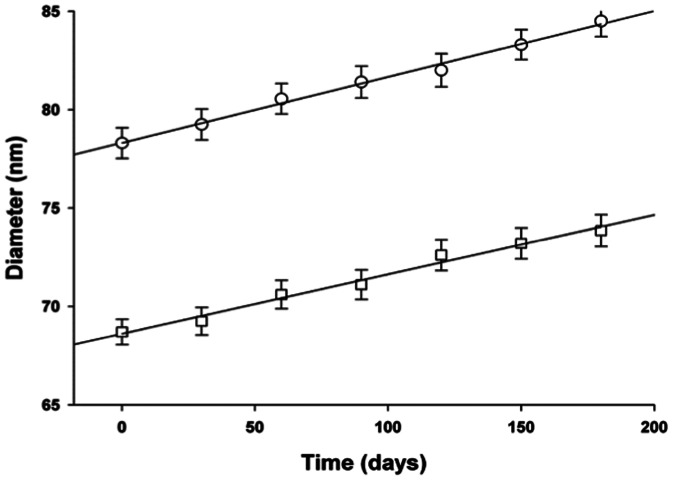
Stability evaluation of the nanoemulsions over time. Key: (□) Unloaded nanoemulsion (R = 0.9965); (○) Riboflavin-5-loaded nanoemulsion (R = 0.9927). Mean of 10 analytical determinations ± standart deviation.

For both, empty and riboflavin-loaded nanoemulsions, the droplet size control up to 180 days showed that there was an increase in the diameter of 7.5%. The increased diameter followed zero-order kinetics, as described by straight-line equations. The data of Figure 1 show that, although the incorporation of the riboflavin into nanoemulsion resulted in an increase in the diameter of the droplets throughout the length of the curve, the lines describing the increase in size during the control of stability have similar slope coefficients.

Equations 1 and 2 describe the increased diameter versus time in unloaded and loaded nanoemulsions, respectively.
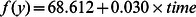
(1)

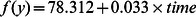
(2)


These results demonstrate that the incorporation of the riboflavin into nanoemulsion did not affect the stability of the system.

Evaluating equations 1 and 2, as well as the data shown in Figure 1, it is possible to estimate that within two years of storage the average diameters of nanodroplets will increase to 89.7 nm for unloaded nanoemulsions and to 102.1 nm for riboflavin-5-loaded nanoemulsion. The concentration of riboflavin in the nanoemulsion protected from light, measured up to 180 days, in the stability test, was not less than 97%. Under these conditions the system remains stable in its colloidal dispersion and the riboflavin content suitable for obtaining the desired effect.

Furthermore, we have found that the drug recovery after filtration through 0.22 µm membrane filter was always greater than 95% of the added drug, regardless the ionic specie of the incorporated riboflavin. The nanoemulsions here presented represent a positively charged system with modified surface by the presence of thermosensitive block copolymer with the ability to increase bioadhesion of the droplets in the corneal epithelium improving the riboflavin permeation to stroma.

### Determination of Riboflavin Absorption Spectra

The absorption of ultraviolet, visible, and infrared radiation depends on the structures of molecules and is typical for each substance. Initially, we determined the absorption spectra of the riboflavin nanoemulsions to verify the wavelength of maximum absorption. Samples of 0.5% riboflavin were diluted with distilled water to obtain values of maximum absorbance less than 1.00.

Sample absorbances were determined using a spectrophotometer (Ultrospec 2000, Pharmacia Biotech, Piscataway, NJ, USA) with quartz cuvettes and the standard 10 mm light path, in the wavelength range from 300 to 600 nm. Distilled water was used as a reference in spectrophotometer readings.

### Experimental Procedures

One-hundred and sixty freshly enucleated eyes (within 6 h of death) from New Zealand rabbits obtained from the local slaughterhouse (Coelho Real – Salto de Pirapora – SP - Brazil) were used for the study (permission was obtained from this slaughterhouse to use these animal parts). The age of the rabbits at the time of death was approximately 90 days. Each specimen underwent slit lamp evaluation, using direct and indirect illumination. Only clear corneas with no edema were used. The specimen was discarded if there was corneal scarring, opacity, or other abnormalities. The corneal thickness, before and after epithelium removal, was measured in all eyes using ultrasound pachymetry (DGH Pachette 3, Exton, PA, USA).

Twenty eyes were not treated with riboflavin solution, but were used to calculate the UVA absorption coefficient at 365 nm of the cornea and to investigate the influence of epithelial removal. Ten of the eyes had the epithelium from the central 8 mm cornea removed using a blunt spatula before measurement.

The remaining 140 eyes were divided into the following groups:

- Standard (n = 10). Eyes with de-epithelialized corneas were soaked with standard hydrophilic riboflavin for 30 minutes. This group was used to evaluate riboflavin absorption characteristics during the standard CXL treatment.
- Standard with intact epithelium (n = 25). Eyes with intact corneal epithelium were soaked with standard hydrophilic riboflavin (0.1% riboflavin-dextran solution) for 30, 60, 120, 180, or 240 minutes (n = 5 at each time point). This group was used to evaluate the possibility of increasing riboflavin absorption by prolonging the exposure time.
- Concentrated with intact epithelium (n = 25). Eyes with intact corneal epithelium were soaked with concentrated hydrophilic riboflavin (1% riboflavin-dextran solution) for 30, 60, 120, 180, or 240 minutes (n = 5 at each time point). This group was used to evaluate whether the increase in concentration of standard riboflavin formulation would increase the uptake through the intact epithelium.
- Riboflavin-5-phosphate nanoemulsion (n = 25). Eyes with intact corneal epithelium were soaked with 0.5% riboflavin-5-phosphate nanoemulsion for 30, 60, 120, 180, or 240 minutes (n = 5 at each time point).
- Riboflavin-base nanoemulsion (n = 25). Eyes with intact corneal epithelium were soaked with 0.5% riboflavin-base nanoemulsion for 30, 60, 120, 180, or 240 minutes (n = 5 at each time point).
- De-epithelialized riboflavin-5-phosphate nanoemulsion (n = 15). Eyes with de-epithelialized corneas were soaked with 0.5% riboflavin-5-phosphate nanoemulsion for 30 minutes. This group was used to evaluate stromal affinity of the riboflavin-5-phosphate amphiphilic formulation.
- De-epithelialized riboflavin-base nanoemulsion (n = 15). Eyes with de-epithelialized corneas were soaked with 0.5% riboflavin-base nanoemulsion for 30 minutes. This group was used to evaluate stromal hydrophilic affinity of the riboflavin-base formulation.

Corneas were soaked with the riboflavin solutions to compensate for the drops running off the globe because of the absence of eyelids. After exposure to the solutions at room temperature, corneas were gently rinsed with balanced salt solution to remove the excess of riboflavin. An 8 mm corneal button was trephined after epithelial integrity had been assured by slit lamp examination. In groups IV and V, the epithelium was removed and absorption measurements were repeated to evaluate riboflavin retention in the epithelium.

### Absorption and Transmittance Evaluation

Corneal transmittance was measured by placing the excised corneas between the UVA light source (X-Link®; Opto Eletronica, Sao Carlos, Brazil) and an external photometer (MRUR-202, Instrutherm, Sao Paulo, Brazil). This photometer was set at 365 nm, with a specific spectrum ranging from 320 to 390 nm. Measurements were taken in controlled ambient light to avoid any external interference.

An 8-mm UVA (365 nm) spot was applied 45 mm from the cornea, with a surface irradiance of 3 mW/cm^2^. Surface irradiance was continuously controlled by a microprocessor in the monitoring system of the device that used an internal power meter.

The absorption coefficient (μ) and transmittance (T) were calculated by the Lambert-Beer formula using the measured corneal thickness (*x*), incident light intensity (I_0_) and transmitted light intensity (I):
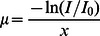


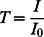



The Lambert-Beer Law expresses the relationship between the amount of absorbed light and the concentration of the solution (i.e., the lower the light transmitted by a solution, the higher its concentration).

### Statistical Analysis

Pairwise comparison between groups was performed using the Mann-Whitney test. All statistical analyses were performed with commercially available software (Stata version 10; StataCorp, College Station, TX). The alpha level (type I error) was set at 0.05. Results are presented as the average ± standard deviation.

## Results

**Figure 2:**
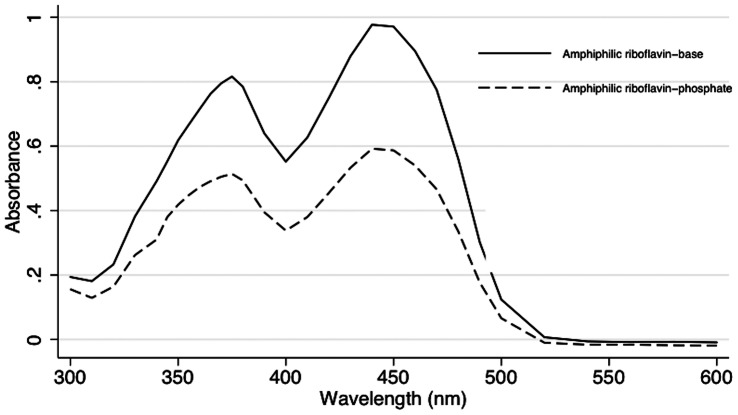
Absorption spectra of riboflavin-5-phosphate nanoemulsion and riboflavin-base nanoemulsion.

The average central corneal thickness measured with ultrasound was 580.0±52.8 µm and 529.0±49.3 µm, before and after epithelium removal, respectively. Both formulations of riboflavin tested showed absorption peaks between 360 and 375 nm (Figure 2). Thus, we used a solid-state device, available in our laboratory, set at 365 nm.

**Figure 3:**
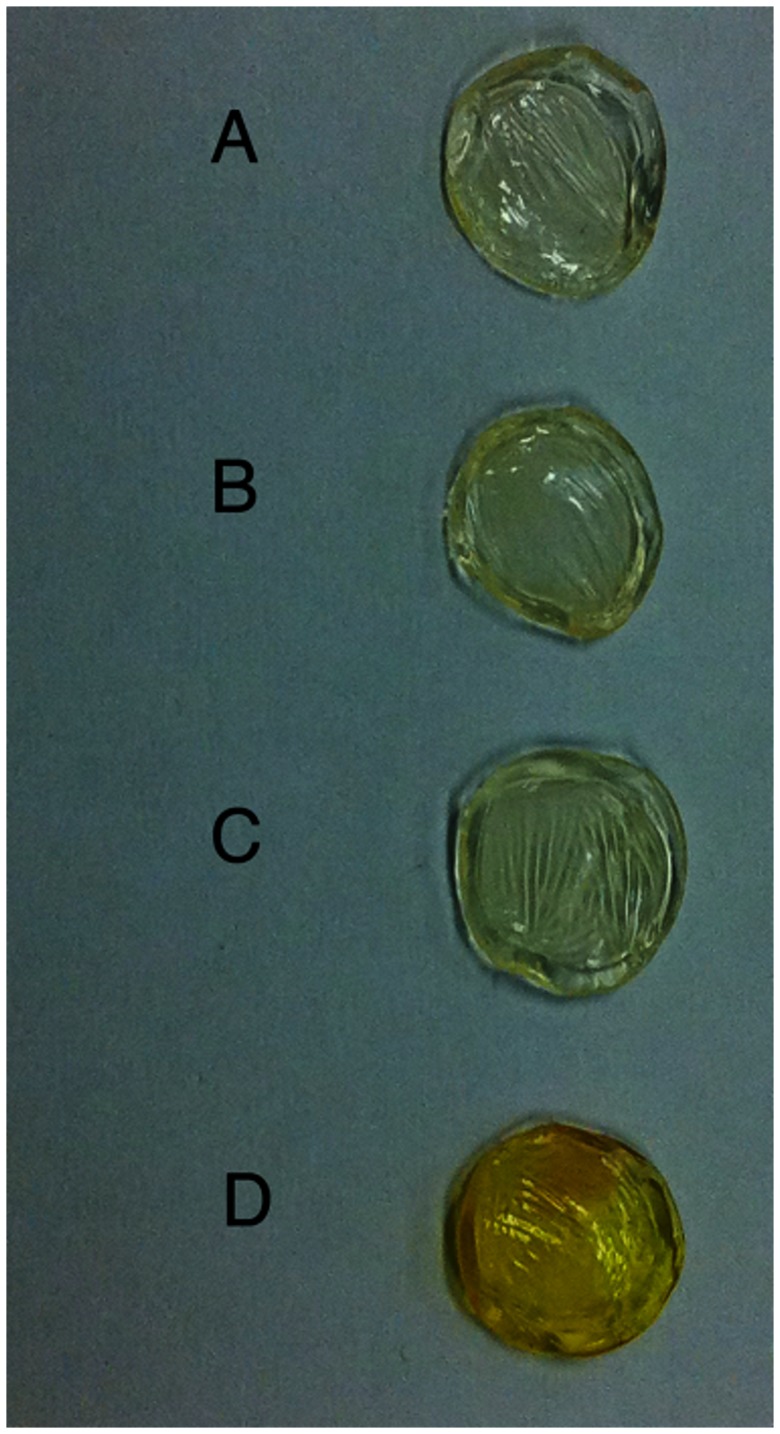
Photograph of the corneal buttons of rabbit eyes of after exposure to different riboflavins. Corneas with intact epithelium (A, B, and C) after exposure to 0.1% riboflavin-dextran solution for 30 minutes (A) and for 240 minutes (B), as well as after exposure to concentrated 1% riboflavin-dextran for 240 minutes (C). De-epithelialized cornea soaked with 0.1% riboflavin-dextran for 30 minutes - standard technique (D). The yellow staining shows that riboflavin diffused into the stroma only when the epithelium was removed.

Absorption and transmittance results are summarized in Table 2 and examples of corneas from the different groups are shown in Figure 3. Removing the epithelium had no significant effect on the absorption characteristics of rabbit corneas that were not exposed to riboflavin. Absorption coefficients were 18.34±0.65 cm^−1^ and 18.53±0.22 cm^−1^, with and without epithelium, respectively (*P* = 0.068).

**Table 2:** Absorption Characteristics of Different Riboflavin Formulations.

Group	Basic Formulation	Riboflavin Type	Riboflavin Concentration	Epithelium	n	Exposure Time (min)	Mean Transmittance (%)	Mean Absorption Coefficient (cm^−1^)
Control A				Intact	10		36.60±1.35	18.34±0.65
Control B				De-epithelialized	10		36.77±0.46	18.53±0.22
Group I	Hydrophilic*	Phosphate	0.10%	De-epithelialized	10	30	17.00±0.51	32.82±0.54
Group II	Hydrophilic*	Phosphate	0.10%	Intact	5	30	34.20±0.37	18.5±0.19
					5	60	34.06±0.51	18.57±0.25
					5	120	33.94±0.64	18.64±0.32
					5	180	33.34±0.35	18.94±0.17
					5	240	32.94±0.25	19.15±0.15
Group III	Hydrophilic*	Phosphate	1%	Intact	5	30	30.86±0.70	20.27±0.38
					5	60	31.20±0.37	20.08±0.21
					5	120	31.00±0.54	20.20±0.29
					5	180	31.14±0.38	20.12±0.21
					5	240	30.74±0.29	20.34±0.16
Group IV	Nanostructured†	Phosphate	0.50%	Intact	5	30	22.86±2.49	25.52±1.91
					5	60	18.38±0.54	29.19±0.51
					5	120	17.54±0.55	30.03±0.55
					5	180	16.74±1.02	30.85±1.07
					5	240	15.00±0.30	32.71±0.38
				De-epithelialized	5	‡	18.54±0.39	31.35±0.44
Group V	Nanostructured†	Base	0.50%	Intact	5	30	31.94±2.61	19.72±1.38
					5	60	26.66±1.37	22.81±0.91
					5	120	23.98±0.82	24.61±0.59
					5	180	24.66±0.94	24.14±0.68
					5	240	24.86±0.67	24.00±0.50
				De-epithelialized	5	‡	25.20±0.67	25.53±0.48
Group VI	Nanostructured†	Phosphate	0.50%	De-epithelialized	15	30	4.26±0.29	58.44±1.20
Group VII	Nanostructured†	Base	0.50%	De-epithelialized	15	30	20.40±1.28	29.47±1.17

* Standard formulation (Dextran T-500 20%, 400 mOsm/ml).

‡ Measurements realized after epithelial removal.

† Microemulsion prepared by sonication.

After exposure to 0.1% riboflavin solution to de-epithelialized corneas for 30 minutes (Group I - Standard), transmittance and absorption coefficients were 17.00±0.51% and 32.82±0.54 cm^−1^, respectively. These values were greatly different from the results observed when the epithelium was left intact (Group II - Standard with Intact Epithelium) using the same exposure time (34.20±0.37%, *p* = 0.002 and 18.50±0.19 cm^−1^, *p* = 0.002) and even after 240 minutes of exposure (32.94±0.25%, *p* = 0.002 and 19.15±0.15 cm^−1^, *p* = 0.002).

Increasing the concentration of the standard hydrophilic solution to 1% (Group III) did not cause similar riboflavin penetration through the intact epithelium, when compared to Group II (*p* = 0.008). After 240 minutes of exposure to the 1% concentrated riboflavin solution, absorption coefficient (20.34±0.16 cm^−1^) and transmittance (30.74±0.29%) were significantly different from the Standard Group (*P* = 0.0021).

**Figure 4:**
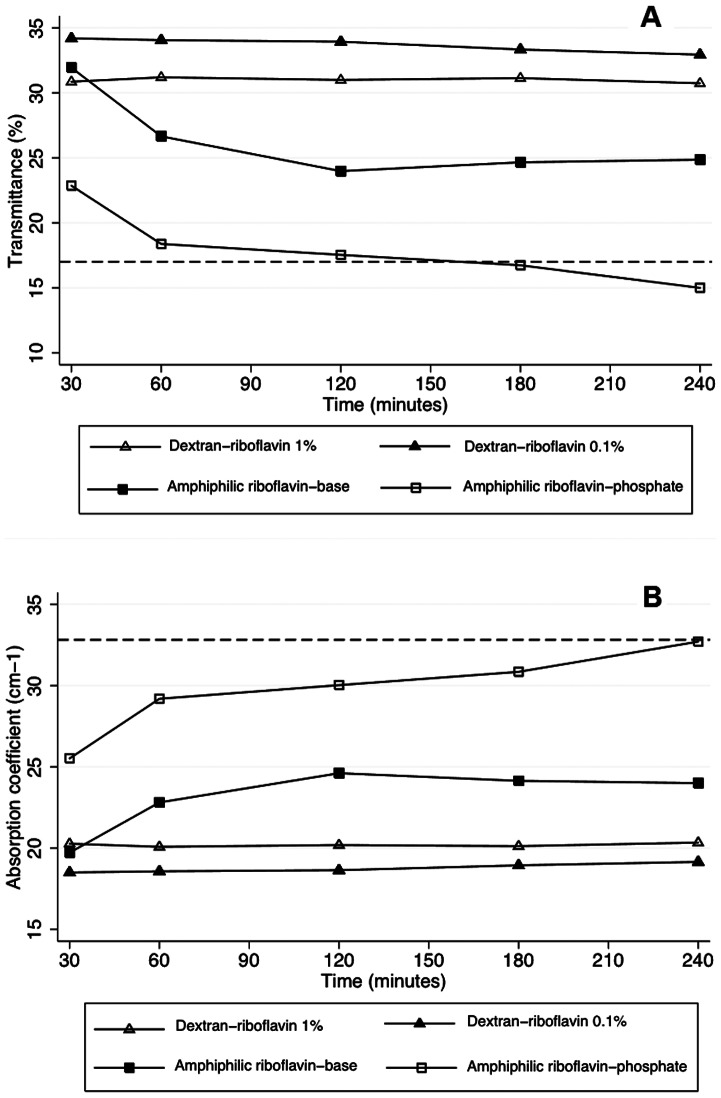
Change of transmittance and absorption coefficient of rabbit corneas in the course of time. Transmittance change (A) and absorption coefficient change (B) of corneas soaked with riboflavin formulations. Dashed lines show group I values (standard treatment).

**Figure 5:**
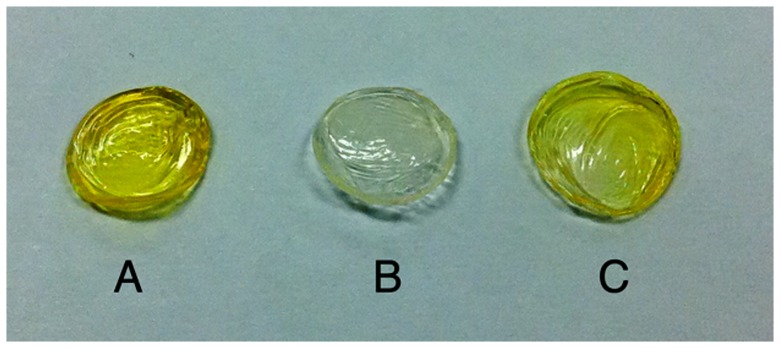
Photograph of corneal buttons of rabbit eyes soaked with riboflavin-5-phosphate nanoemulsion or standard 0.1% riboflavin. Cornea with intact epithelium after exposure to 0.5% riboflavin-5-phosphate nanoemulsion for 240 minutes (A). Cornea with intact epithelium after exposure to the standard 0.1% riboflavin solution for 240 minutes (B). De-epithelialized cornea exposed to 0.1% riboflavin solution for 30 minutes - standard technique (C).

Figure 4 shows the transmittance and absorption coefficients of corneas exposed to the riboflavin nanoemulsions (Groups IV and V). In Group IV (0.5% riboflavin-5-phosphate), transmittance was not different to the standard technique after 120 minutes (*p* = 0.08) and became greater at 240 minutes (*p* = 0.002). Macroscopically, a homogenous yellow stain representing riboflavin diffusion into the stroma was observed (Figure 5). The absorption coefficient was not statistically different from the standard group after 240 minutes (*P* = 0.54).

The absorption coefficient (*p* = 0.0076) and transmittance (*p* = 0.0076) of the riboflavin-base nanoemulsion (Group V) were higher than the standard formulation in an intact epithelium (Group II). However, after 240 minutes, the values were still significantly different than the standard protocol (Group I; *p* = 0.002 for absorption coefficient and transmittance).

Measurements taken on the same corneas of Group IV and V after epithelium removal showed that both nanoemulsions were able to diffuse into the corneal stroma. Corneal absorption measurements were greater in these corneas compared to the standard group with epithelium (Group II). Mean absorption coefficient and transmittance were 18.54±0.39% (*p* = 0.0086) and 31.35±0.44 cm^−1^ (*p* = 0.0086), for the riboflavin-5-phosphate nanoemulsion and 25.20±0.67% (*p* = 0.0086) and 25.53±0.48 cm^−1^ (*p* = 0.0086), for the riboflavin-base nanoemulsion. Riboflavin-5-phosphate nanoemulsion diffused into the stroma better than the riboflavin-base nanoemulsion (*p* = 0.008 for absorption coefficient and transmittance).

When eyes with debrided corneas were exposed to the nanoemulsions (Groups VI and VII), riboflavin-5-phosphate had a greater stromal affinity than its base counterpart. After 30 minutes, the transmittance was 4.26±0.29% and the absorption coefficient was 58.44±1.20 cm^−1^. These values were statistically different than the riboflavin-base values of 20.4±1.28% (*P* = 0.008) and 29.47±1.17 cm^−1^ (*P* = 0.008), respectively.

## Discussion

During CXL, stromal diffusion of riboflavin is necessary because it is the basis of CXL safety and effectiveness. Riboflavin is not only important as a photosensitizer, but also because it shields UVA light from damaging the underlying ocular structures such as the endothelium, lens, and retina. UVA radiation decreases as it passes through the cornea, because riboflavin in the cornea absorbs the highest amount of UVA, preventing high doses of this radiation from reaching the posterior ocular structures [36], [37]. Until now, epithelium removal is the standard method to facilitate penetration of riboflavin into the corneal stroma. In the present study, we describe a new option to achieve stromal diffusion of riboflavin without removal of the epithelium and without the use of benzalkoniun chloride.

Our group has reported that the epithelium prevents the penetration of the standard 0.1% riboflavin-dextran solution, but apparently does not block UVA radiation [4]. Although the epithelium can potentially block some UV radiation, its absorption is greatest for wavelengths lower than 300 nm [38]. Therefore, efficacy of CXL should not be decreased in the transepithelial technique with the nanostructured riboflavin presented in this study because both UVA radiation and riboflavin can penetrate the corneal stroma.

After applying riboflavin in dextran solution to the de-epithelialized rabbit corneas for 30 minutes (standard CXL protocol), the absorption coefficient was 32.82±0.54 cm^−1^ and the resulting transmittance was 17.00±0.51%. Previous reports presented higher levels (63 cm^−1^ for rabbit corneas [36], 51.46 cm^−1^ for porcine corneas [11], and 56.36 cm^−1^ for human corneas [11]). This may be explained because the riboflavin film was included in these measurments. With removal of the riboflavin film from the stromal surface, our values are consistent with previous reports [11], [22]. Using 0.5% riboflavin-5-phosphate nanoemulsion for 4 hours in corneas with intact epithelium, we observed comparable absorption values to the standard de-epithelialized CXL treatment. This indicates that, although the exposure time was long, this new riboflavin nanoemulsion was able to penetrate efficiently the epithelium of rabbit corneas.

Permeability is dependent on the chemical affinity and the size of the diffusing molecule. Our experiments show that riboflavin penetration across an intact epithelium was possible, however, 240 min of exposure was required, probably due to the high molecular weight of riboflavin (376.32 g/mol). Recently, the results of another study on the same topic illustrated the utility of using a synthetic peptide (NC-1059) to allow riboflavin to pass through the intact epithelium, reaching concentrations almost as high as if epithelium had been scraped off first [27]. Study data demonstrated that NC-1059 transiently opened the epithelial intercellular tight junctions by forming a nonselective ion channel that reversibly alters epithelial barrier function without killing corneal epithelial cells. In the future, combination of the synthetic peptide described by Zhang et al with the present riboflavin-5-phosphate nanoemulsion might allow a faster diffusion of riboflavin through the intact epithelium [27].

Although topical instillation of drugs is very popular in ophthalmology, the cornea is an effective barrier to hydrophilic drugs such as the standard riboflavin solution. Corneal epithelium is lipophilic and has annular tight junctions that completely surround and effectively seal the superficial epithelial cells. Many methods have been developed to enhance transepithelial absorption of hydrophilic therapeutic agents. One approach is to modify the physicochemical property of drugs by nanotechnology.

Colloidal delivery systems based on microemulsions or nanoemulsions are increasingly being utilized in the food and pharmaceutical industries to encapsulate, protect, and deliver bioactive components [28]. The small size of the particles in these kinds of delivery systems (*r*<100 nm) means that they have a number of potential benefits for certain applications, such as enhanced long-term stability; high optical clarity and increased bioavailability. Nanoemulsions are potent drug delivery vehicles for ophthalmic use due to their sustained effect and high ability of drug penetration into the deeper layers of the ocular structure and the aqueous humor [39]. To date, a range of drugs has been formulated in a nanoemulsion for ophthalmic application, including dorzolamide hydrochloride [40] and indomethacin [41].

The role of penetration enhancers played by the amphiphilic components of the nanoemulsion and the internal mobility of the drug within the vehicle also contribute to its overall performance in drug delivery. Amphiphile describes chemical compounds containing both hydrophilic and lipophilic properties. Amphiphilic molecules have a polar, water-soluble group attached to a non-polar, water-insoluble hydrocarbon chain. An amphiphilic drug has greater penetration through both the lipophilic epithelium and the corneal stroma, which has hydrophilic characteristics. Based on this concept, we developed two amphiphilic formulations (nanoemulsions) using different riboflavins (phosphate and base). Riboflavin-5-phosphate is a hydrophilic salt that can be delivered to the stroma using the amphiphlic vehicle, despite the lipophilic characteristics of the epithelium. Because the riboflavin-5-phosphate has hydrophilic properties, it is retained at a greater concentration in the stroma. However, although the riboflavin-base is able to pass through the epithelium, it cannot be retained in the stroma, due to the lipophilic characteristics of the salt. Our study showed that the riboflavin-5-phosphate nanoemulsion had a better diffusion into the stroma of corneas with intact epithelium than the riboflavin-base.

Transepithelial CXL has been suggested as an alternative approach to overcome the inherent disadvantages of epithelial scrapping. However, none of the previous proposed methods has proven to saturate the stroma comparably to the standard CXL protocol. As the absorption values of the new riboflavin-5-phosphate nanoemulsion at 240 minutes were similar to those obtained by corneas with removal of the epithelium in the standard CXL protocol, transepithelial approach using this formulation might be an alternative approach to overcome limitations of previously proposed methods.

One of the limitations of this study is the use of dissected rabbit eyes. It is not possible to obtain a homogeneous sample size using human donor corneas. We decided to use rabbit corneas because of their similarities with human corneas. Porcine eyes were not chosen because of the thick stroma and epithelium, which could result in a low value for the absorption coefficient. Previous studies have demonstrated that bovine freshly enucleated eyes can be used to evaluate corneal permeability [26]. Although there is no scientific evidence of which is best animal model for the study of corneal permeability, the thickness seems to play an important role. When considering the treatment of thin human corneas, as in keratoconus, the rabbit model may be suitable [42]. To limit corneal edema and the resulting increase in corneal thickness, only fresh eyes less than 6 h after euthanization were used for measurements. However, further research on the safety and efficacy must be done before using this drug in patients. Although, riboflavin absorption into to the stroma is an essential step for CXL treatment and its evaluation is essential before conducting clinical studies; further experiments are needed to evaluate the mechanical characterization of cross-linked corneas using the new riboflavin nanoemulsion. Further studies should evaluate safety and effectiveness of the use riboflavin drops for a few hours before being treated with 30 minutes of UVA irradiation.

In this study we demonstrated that riboflavin nanoemulsion could be delivered into the stroma in corneas with intact epithelium. We were able to obtain stromal concentrations of riboflavin similar to the standard CXL technique in de-epithelialized corneas. The riboflavin-5-phosphate nanoemulsion more efficiently diffused into the stroma than the riboflavin-base nanoemulsion. To the best of our knowledge, this is the first study using a riboflavin nanoemulsion to achieve epithelial permeation of the drug. The present study may have significant implications for the refinement of the CXL technique. With the development of riboflavin nanoemulsion, the transepithelial technique may in a near future become a real option in CXL treatment.
